# Analgosedation in paediatric severe traumatic brain injury (TBI): practice, pitfalls and possibilities

**DOI:** 10.1007/s00381-017-3520-0

**Published:** 2017-09-06

**Authors:** N. Ketharanathan, Y. Yamamoto, U. Rohlwink, E. D. Wildschut, M. Hunfeld, E.C.M. de Lange, D. Tibboel

**Affiliations:** 1 Intensive Care and Department of Paediatric Surgery, Erasmus Medical Center, Sophia Children’s Hospital, Rotterdam, The Netherlands; 2 Erasmus MC-Sophia Children’s Hospital, PO Box 2060, 3000 CB Rotterdam, The Netherlands; 3 Leiden Academic Center for Drug Research, University of Leiden, Leiden, The Netherlands; 4 Division of Neurosurgery, Red Cross War Memorial Children’s Hospital, University of Cape Town, Cape Town, South Africa

**Keywords:** Traumatic brain injury, Paediatric, Analgesia, Sedation, Pharmacology

## Abstract

Analgosedation is a fundamental part of traumatic brain injury (TBI) treatment guidelines, encompassing both first and second tier supportive strategies. Worldwide analgosedation practices continue to be heterogeneous due to the low level of evidence in treatment guidelines (level III) and the choice of analgosedative drugs is made by the treating clinician. Current practice is thus empirical and may result in unfavourable (often hemodynamic) side effects. This article presents an overview of current analgosedation practices in the paediatric intensive care unit (PICU) and addresses pitfalls both in the short and long term. We discuss innovative (pre-)clinical research that can provide the framework for initiatives to improve our pharmacological understanding of analgesic and sedative drugs used in paediatric severe TBI and ultimately facilitate steps towards evidence-based and precision pharmacotherapy in this vulnerable patient group.

## Introduction

The main goal of (paediatric) TBI treatment is the provision of supportive care to guarantee adequate cerebral perfusion to meet cerebral metabolic demands and limit secondary brain injury [1, 2]. Appropriate management is critical as paediatric TBI still causes major morbidity and mortality worldwide [3, 4]. One of the cornerstones in severe paediatric TBI treatment is providing analgesia and sedation; however, current research efforts have not provided any level 1 evidence to support a specific regimen. This has resulted in the international 2012 TBI guideline recommendation that: ‘the choice of and dosing of sedative, analgesics …. should be left to the treating physician..’ [5]. This leads to heterogeneous analgosedation prescribing practices based on local expertise and guided by (hemodynamic) side effects which can lead to both over and under sedation. Furthermore, it seems that exposure to certain analgosedation drugs might lead to neurotoxic injury and could be associated with unfavourable outcome in the long term (e.g. radiographic decrease in brain mass, learning disabilities), especially in the developing paediatric patient [6–8]. Although it is difficult to differentiate between the sequelae of primary vs. secondary brain injury, such observations necessitate better pharmacological understanding to achieve both effective and safe analgesia and sedation. To that end, it is important to gain insight into the pharmacokinetics (PK; i.e. ‘what the body does to the drug’) and the resulting pharmacodynamics (PD; i.e. ‘what the drug does to the body’) of frequently used analgosedative drugs. It should be noted that plasma PK may differ substantially from brain target site PK due to the functionalities of the blood-brain-barrier (BBB) and the blood-cerebrospinal fluid barrier (BCSFB) [9] and information on drug target site distribution is essential to understand PK-PD relationships in different TBI disease states. Collaboration between preclinical and clinical research is essential in this respect. Techniques such as high-frequency multimodal neuromonitoring, including cerebral microdialysis, combined with advanced PK-PD modelling are promising tools [10, 11]. The physiologically based (PB) pharmacokinetic (PBPK) modelling approach is particularly useful as it allows translation from animals to humans. Measurements that cannot be performed (or only highly limited) in humans can be performed in animals, and the resulting data can be physiologically scaled to predict PK in humans. This is imperative to fully understand how drugs behave in the body and vice versa. This improved pharmacological understanding will facilitate development of more patient-specific treatment strategies in a heterogeneous condition like paediatric TBI, which requires a tailored approach to achieve effective and safe therapy. This narrative review aims to provide an overview of current practice and its potential pitfalls and discusses possibilities of developing more evidence-based analgosedation practices in paediatric TBI.

## Methods

A literature search was performed using search strategies with MeSH terms and synonyms of ‘brain injury’, ‘analgesia’, ‘sedation’ and ‘children’ with the search databases Embase, Ovid, Cochrane Central, Web of Science and Google Scholar. A total of 1160 potential references were found that were reviewed by the primary author for relevance based on the aim to provide a pharmacological overview of paediatric severe TBI analgosedation practice and principles. Additional references were added by cross-reference check. It must be emphasized that the 2012 international guidelines for paediatric TBI management remain the mainstay of treatment recommendations [5]. This article provides additional pharmacological background information and highlights novel approaches in pharmacological research.

## Practice

The application of analgesia and sedation in the stabilisation and (supportive) treatment of paediatric severe TBI occurs in various settings from the scene of the accident, emergency department, perioperatively and after admission and stay in the paediatric intensive care unit (PICU). The primary aim for administering analgosedation is to lower cerebral metabolic demand which can be increased by anxiety, stress and pain induced by the primary trauma and further exacerbated by noxious stimuli such as (invasive) procedures and invasive ventilation [2, 12–14]. This article will focus on analgosedation practices in the PICU. The ideal properties of analgosedation drugs in paediatric severe TBI would be to induce an immediate analgesic and/or sedative effect without negatively affecting hemodynamic stability or intracranial pressure (ICP) and have rapid offset of action to enable neurological examination. The compound should also be safe in the long term with respect to neurotoxicity. Many frequently used compounds offer some of the aforementioned characteristics, but none have so far demonstrated all favourable elements. Perhaps, this is not possible given the physiological and developmental differences between patients from ages 0 to 18 years and the heterogeneous nature of brain injury itself.

Most PICUs adopt a TBI protocol based on international guidelines, consensus and local variations in practice. The provision of sedation and analgesia is subdivided in so-called first and second tier therapies in a step-up approach to control ICP and maintain adequate cerebral perfusion pressure (CPP) [5, 15]. Adherence to local protocols with respect to analgosedation varies based on personal preference and patient-specific factors. A survey in the USA explored physician agreement with published recommendations and guidelines for paediatric TBI showed a 91% agreement with first-tier sedation recommendations and a 89% agreement with the use of barbiturates as second tier therapy. However, no specification was provided as to which agents were used when providing analgosedation [16]. One study, which audited its analgosedation practice, reported a 64% adherence to their written protocol with patient-specific adjustments in 30% of these cases [17]. This illustrates the variation in analgosedation practice for paediatric TBI, which is not surprising given the lack of evidence-based regimen.

Analgesic compounds described in the literature for paediatric severe TBI are opioids (Fentanyl, Remifentanil, Morphine), Acetaminophen and Metamizole [12, 17–20]. Sedative compounds include benzodiazepines (Midazolam, Lorazepam, Diazepam), Ketamine, Propofol, Dexmedetomidine and Etomidate [12, 14, 17, 18, 21]. Barbiturates (Thiopental and Pentobarbital) are part of second tier therapeutic strategies for lowering refractory intracranial hypertension under electroencephalogram (EEG)-monitoring to ascertain burst-suppression [2, 12, 14, 18, 22–24]. Tables 1 and 2 provide an overview of the pharmacological properties of the various analgosedative drugs (only Food and Drug Administration (FDA) approved drugs included).

**Table 1:** Overview analgesic drugs in paediatric severe traumatic brain injury

Class	Medication	Mode of action	Dosage	T ½	Side effects	PK points of interest
Opioid	Morphine	Opiate receptor agonist	Bolus 0.05–0.1 mg/kg	2–4 h	Respiratory depression, hypotension, urinary retention, vomiting, constipation, pruritus	Active metabolite morphine-6-glucuronide (M6G)
			Range 0.01–0.04 mg/kg/h			Direct interaction with opioid binding sites in brain
						Active efflux mechanism in brain (P-glycoprotein and multidrug resistance-associated protein (MRP)) and active influx transporter (undefined)
	Fentanyl	Opiate receptor agonist: inhibition of nociceptive neurotransmitters	Bolus 1–2 mcg/kg	7 h	Sedation, respiratory depression, hypotension, muscle rigidity (thorax), nausea	High lipophilicity leading to easier penetration CNS. Not subjected to active transport across the BBB
			Range 1–2 mcg/kg/h			
	Remifentanil	Opiate (specific) mu-receptor agonist	Bolus 0.25–1 mcg/kg	1–20 min	Respiratory depression, muscle rigidity, hypotension, nausea, pruritus	Direct interaction with opioid binding sites in brain
		Ultra-rapid onset and offset of action	Range 0.1–2 mcg/kg/min			
Other	Acetaminophen	Inhibition of cyclooxygenase (COX) enzymes involved in prostaglandin synthesis	Loading dose 20 mg/kg i.v.	1–4 h	Risk of hepatic necrosis and failure in prolonged or high dosage	Blockage of COX enzymes in the CNS
			Maintenance 60 mg/kg/dg in 4 doses (maximum 1 g per dose)			

The abovementioned dosage suggestions are for children aged 0–18 years of age, with the exception of neonates (<1 month old)

*BBB* blood-brain-barrier, *COX* cyclooxygenase, *CNS* central nervous system, *i.v.* intravenous, *MRP* multidrug resistance-associated protein, *PK* pharmacokinetic

**Table 2:** Overview sedative drugs in paediatric severe traumatic brain injury

Class	Medication	Mode of action	Dosage	T ½	Side effects	PK points of interest
Benzodiazepine	Midazolam	GABA receptor agonist	Bolus 0.05–0.2 mg/kg	5.5 h (±3.5 h)	Sedation, respiratory depression, hypotension, bradycardia, (paradoxical) emergence delirium	Metabolized to active metabolites 1- and 4-hydroxy-midazolam
			Range 0.05–0.5 mg/kg/h			Lipophilic
Barbiturate	Pentobarbital	GABA receptor agonist	Bolus 5 mg/kg	5–50 h (dose dependant)	Sedation, respiratory depression, hypotension, laryngospasm, anaphylactic reactions have been reported	Lipophilic
			Range 1–2 mg/kg/h			
	Thiopental	GABA receptor agonist	Range 12,5 mg/kg/h (starting dose for 6 h), then 5 mg/kg/h and taper to 3 mg/kg/h depending on EEG and plasma concentration	3–8 h	Sedation, respiratory depression, hypotension, skin reactions (due to histamine release), laryngo- and bronchospasm	Lipophilic
Other	Propofol	GABA receptor agonist	Bolus 1–2 mg/kg	2–10 min (initial distribution phase)	Hypotension, respiratory depression, hypertriglyceridemia, PRIS	Lipophilic
			Range (not recommended but reported up to 4 mg/kg/h)			
	Dexmedetomidine	Selective alpha-2-adrenoreceptor agonist	Range 0.1–2 mcg/kg/h (loading dose 0.25 mcg/kg optional)	2 h	Hypertension, bradycardia	Lipophilic
	Ketamine	*N*-methyl-D-aspartate receptor antagonist	Bolus 1–2 mg/kg	2.5–3 h	Laryngospasm, tachycardia, dysphoria	Lipophilic
	Etomidate	GABA receptor agonist	Bolus 0.3–0.5 mg/kg	75 min	Adrenal insufficiency, hypertension, laryngospasm, dyskinesia	Lipophilic

The abovementioned dosage suggestions are for children aged 0–18 years of age, with the exception of neonates (<1 month old)

*EEG* electroencephalogram, *GABA* gamma-aminobutyric acid, *PK* pharmacokinetic, *PRIS* propofol infusion syndrome

Most commonly, a combination of an analgesic and sedative drug is prescribed as a continuous infusion with a step-up approach in dosing based on clinical effect (often a combination of neurological exam, ICP-threshold, and pain and sedation scoring systems). The sedative effect of most analgesic compounds during dose escalation is often an added benefit in ICP control [14]. Additional bolus medication is common to combat prolonged ICP elevation and determine if pain could be a possible cause thereof [14]. Reports indicate that Fentanyl and Midazolam (separately or in conjunction) are mostly used as bolus analgosedation. Various studies have been performed to clarify which bolus medications are most effective to lower high ICP and present conflicting and sometimes paradoxical results [25]. The benefit of Ketamine for episodic use during potential noxious stimuli (e.g. endotracheal tube suctioning) is that it does not decrease blood pressure and could be a valuable adjuvant for interventions [26]. However, historically, this compound has been avoided as it was associated with an increase in ICP [27–29]. Recent paediatric studies using (racemic) Ketamine as bolus medication for episodes of sustained ICP elevation demonstrate its effect in lowering intracranial hypertension [18]. Adult studies show similar findings and support its use when combined with Midazolam [30]. Remifentanil has been shown to be an effective analgesic drug (mostly demonstrated in the adult critical care setting) due to its rapid onset and offset of action enabling frequent, serial neurological examinations [19, 20, 31]. Barbiturates (Thiopentone and Pentobarbital) are implemented as second tier therapy to control refractory intracranial hypertension and have been shown to be effective in reducing ICP in the paediatric population in 30–52% of patients [23, 32]. Dexmedetomidine is gaining increasing interest as a sedative agent in the PICU due to its limited side effects although the majority of publications are based on postoperative cardiac patients. It has been approved by the FDA for sedation <24 h in adults with invasive ventilation and there are reports of its use in the PICU for severe TBI patients (who went on to have good neurological outcomes) and it has been reported to have a opioid-sparing effect in PICU patients requiring sedation [21, 33]. Finally, a brief word on neuromuscular blockade (NMB) is warranted as this is combined with analgosedation in first-tier strategies to reduce energy expenditure (e.g. shivering) and improve mechanical ventilation synchronization. Again no class I evidence for NMB is available but it is imperative that at the same time continuous electroencephalography is implemented when continuous NMB infusion is used to determine the presence of posttraumatic seizures [5, 14, 34].

At this point, it needs to be noted that ‘efficacy’ of analgosedation is often titrated solely on the effect of ICP- and CPP-control and clinical signs of agitation. However, there is increased awareness that there are additional factors which need to be considered to develop a holistic picture of risk factors for secondary injury that could serve as pharmacodynamic (PD) markers in analgosedation studies. Multimodal neuromonitoring seems especially important when defining clinical parameters of therapeutic efficacy. PD markers of effective drug therapy could include cerebral oxygenation (PbtO2), cerebral metabolic state (cerebral microdialysis yielding information on lactate and pyruvate) and brain activity as monitored by continuous electroencephalogram (EEG) [10]. Furthermore, it might be beneficial to differentiate between patients with or without intact cerebral autoregulation as certain drugs could actually induce an ICP increase (and CPP decrease) when cerebral autoregulation is preserved [35, 36]. In this respect, the time from initial injury can also be an important factor, resulting in a different response of the brain to analgosedative drugs over time.

## Pitfalls

The potential negative effects of commonly used analgosedative drugs can be subdivided into short- and long-term pitfalls. Short-term pitfalls mostly refer to undesirable hemodynamic side effects, i.e. a decrease in mean arterial blood pressure (MAP) resulting in a decrease in cerebral perfusion pressure (CPP), which can further exacerbate secondary brain injury. Potential pitfalls based on specific drug or patient characteristics are summarized in the following paragraph. A more extensive overview of side effects is provided in Tables 1 and 2.

Continuous infusion of Propofol is associated with Propofol infusion syndrome (PRIS), which is characterized by the onset of metabolic acidosis, rhabdomyolysis, acute renal failure, cardiac dysthythmias and hyperlipidemia [37, 38]. Although there is evidence that continuous Propofol infusion <4 mg/kg/h for periods >48 h can be safe [39, 40], the cost-of-error is deemed so great that the FDA recommends withholding from continuous Propofol infusion in paediatric TBI [41]. As for bolus medication, Propofol is sometimes briefly used as an adjuvant for procedural sedation. However, it is noteworthy that delayed posttrauma administration of Propofol could be associated with induction of apoptosis due to increased expression of the P75 neurotrophin receptor, sensitizing the brain for neurotoxic effects in juvenile neurons [42]. Etomidate could be useful as bolus therapy for intracranial hypertension episodes due to its favourable hemodynamic profile [43]; however, its use is often restricted to intubation settings due to the association with adrenal insufficiency [2]. Thiopental can potentially cause life-threatening electrolyte imbalances, especially regarding potassium [44]. Dexmedetomidine has been reported to induce hypertension at high-dose infusion (>4 μg/kg/h) for several hours [45]. Other reports have noted the occurrence of bradycardia, which is more pronounced when this agent is combined with other medications that yield a negative chronotropic effect [21]. Metamizole is frequently used as a non-opioid first-line analgesic for adults in many countries but there are still many controversies about potential risks (like agranulocytosis), leading to a ban by the FDA. A recent systematic review and meta-analysis showed that Metamizole is safe (no reports of agranulocytosis) for short-term use in hospital [46]. Although its role in paediatric analgesia is unclear, it is reportedly used in certain countries [17]. Various studies have demonstrated that opioids could potentially increase ICP in TBI patients with preserved cerebral autoregulation in conjunction with systemic effects of mean blood pressure decrease. This effect was most pronounced for Fentanyl [36, 47]. Furthermore, a recent study on the effectiveness of pharmacological therapies for intracranial hypertension demonstrated that Fentanyl was significantly associated with frequent treatment failure when compared to Pentobarbital and hypertonic saline [48]. Fentanyl and Midazolam as bolus therapies for intermittent intracranial hypertension have been associated with ICP increase and CPP reduction [35].

Spreading depolarizations present a potential threat to functional neurons, resulting in necrosis or degeneration and possibly poor outcome after brain injury. Analgosedation acts by influencing neuronal homeostasis and has been investigated for preventing or propagating spreading depolarizations. One study found that *N*-methyl-D-aspartate (NMDA) receptor agonists (such as Ketamine) led to less spreading depolarizations, but Midazolam increased the amount of spreading depolarization clusters. These findings need to be investigated further but provide an interesting angle into pitfalls and possibilities of these drugs [49]. Finally, it must be emphasized that the severe TBI patient is often subject to polypharmacy (i.e. multiple drugs being prescribed concurrently). It is important to be aware of drug-drug-interactions, which can lead to subsequent undesirable effects. For example, the proton-pump inhibitor Esomeprazole is frequently prescribed in critically ill patients. It has been reported that prescribing Esomeprazole increases the volume of distribution and half-life of Thiopentone, which needs to be taken into account when aiming for certain target concentrations [50].

Long-term pitfalls have been described in the context of (major) withdrawal symptoms and delirium, which obscure adequate neurological evaluation. This suggests not only the need for dose escalation in the acute phase of patient stabilisation but also the development of guidelines to optimally taper analgosedation afterwards [51]. Validated tools to assess withdrawal symptoms (which can already occur after 5 consecutive days of opioid and/or benzodiazepine administration) and delirium in the PICU are the Withdrawal Assessment Tool version 1 (WAT-1) and Sophia Observation withdrawal Symptoms-Paediatric Delirium scale (SOS-PD) [52, 53].

Pharmacogenetics can be considered part of patient-specific pitfalls. Important examples are so-called ABCB1 polymorphisms, which encode for P-glycoprotein transporter that is responsible for morphine efflux over the blood-brain barrier. This can result in different cerebral morphine concentrations and associated (prolonged) effect. The latter has mostly been investigated in the context of prolonged respiratory depression after opioid administration during elective procedures [54, 55].

Finally, there are concerns about the potential neurotoxic apoptotic effects of certain drugs, especially in the developing brain. Animal studies have demonstrated mechanisms by which analgosedative agents of different classes (which are also used in the PICU for TBI) trigger upregulation of apoptotic cerebral cascades (i.e. blocking of glutamate NMDA receptors, activation of gamma-aminobutyric acid (GABA), receptors or blocking of voltage gated sodium channels). This results in inactivation of cell survival signalling proteins and activation of inflammatory cytokines [7]. Furthermore, anaesthetic opioid exposure in animals showed a possible neurotoxic effect as demonstrated by elevated levels of S100B, a biomarker of brain injury [56]. It is of interest that these effects in animals and cultured neuronal cells were dose dependant and dependant on the maturity of neuronal tissue [57]. Studies in preterm infants exposed to Fentanyl suggest that a higher cumulative dose correlated with an increased incidence of cerebellar injury and lower cerebellar size when compared at term equivalent age [6]. However, the results from paediatric general anaesthesia studies evaluating long-term neurodevelopmental outcome are more difficult to interpret as these are heterogeneous populations often with a pre-existing condition (e.g. congenital defect) giving rise to an a priori increased risk of deficits in neurological development [8, 58]. Of course extrapolation of preclinical and paediatric anaesthetic clinical study findings to analgosedation practices in paediatric TBI should be done with caution, despite the use of similar compounds. However, these observations are important as they emphasise the necessity to search for both effective and safe analgosedation dosing strategies in paediatric TBI.

## Possibilities

To date, current approaches have not been able to conclusively yield the desired answers, i.e. which drugs are safe and effective to prescribe in (paediatric) TBI? The high failure rate in CNS drug development and research can in part be attributed to the fact that ‘golden standard’ approaches, such as randomized controlled trials (RCTs) and comparative effectiveness designs, fail to take into account the heterogeneity of the disease process and drug- and patient-specific characteristics. In particular, the unique functionality of the blood-brain and blood-cerebrospinal (CSF) barrier, and therewith drug transport into and out of the brain, are not elucidated. Thus, drug target site concentration (e.g. cerebral drug concentrations) are unknown. Information on the target site concentrations is fundamental in understanding drug action. Therefore, assessing target site pharmacokinetics is the primary step before attempting to study the resulting pharmacodynamics [9, 11, 59].

Bottlenecks in pharmacological research, especially in children, are ethical concerns about performing invasive studies of compounds with unproven benefit in minors, and difficulty obtaining adequate sample size to provide definitive answers. Another obstacle in paediatric drug research is the limited number and volume of blood or cerebral fluid samples which can be obtained per patient due to smaller circulating volumes. This is where ‘bench-meets-bedside’ research can be of value. Animal-based PBPK models can be translated to humans and thereby serve as templates of what happens to a drug in the body under various clinical conditions. After translation of the animal PBPK model to humans, full drug concentration PK profiles in human brain can be predicted. The PBPK human model can be validated by comparing predicted drug concentration values to a number of observed drug concentrations in the human CNS (e.g. CSF and brain extracellular fluid from microdialysis monitoring). Especially for paediatric PK analysis, obtaining individual plasma PK is crucial to predict a drug concentration time profile in the CNS due to the various developmental differences that have an impact on PK profiles. Collecting sufficient human samples can be challenging, but ‘sparse sampling population-based PK (PPK) analysis’ is an innovative statistical technique that can be used to solve this challenge. Practically, this model requires only a limited number of samples per patient. The samples of various patients are then used as a single ‘population’ (after corrections of covariates such as age, weight, renal function) providing enough data points to compile drug-specific pharmacokinetic time profiles under different clinical conditions and developmental differences. In other words, the combination of PBPK and PPK modelling allows prediction of drug concentrations at various target sites (e.g. blood plasma, brain extracellular fluid) at different dosing regimens for each paediatric patient. It has been enlightening that drug plasma concentrations do not represent cerebral drug concentrations, highlighting the unique properties of the blood-brain and blood-cerebrospinal fluid barrier [11, 59–61].

Although focus must shift ‘back to basics’ with regard to improved pharmacological understanding of frequently used analgesics and sedatives, this does not mean observations from the clinical field should be dismissed. For example, comparative effectiveness research could provide direction as to which drug compounds warrant further PK-PD research. Finally, pharmacology is dynamic, complicated and an indispensable part of clinical practice. It is imperative we understand basic principles but also cooperate with experts in the field to optimize our treatment. In this respect, a clinical pharmacologist or hospital pharmacist can be of great value to point out significant drug interactions and provide alternative options as well as dose adjustments based on clinical status. It is becoming increasingly clear that a multifactorial, complex disease like (paediatric) severe TBI warrants a multidisciplinary approach, also as regards pharmacology.

## Conclusion

Analgosedation is a crucial part of severe (paediatric) TBI management but evidence-based guidelines are not available leaving the choice of analgesic and sedative drugs to the treating physician. *Practice* is heterogeneous and leads to serious concerns about efficacy and safety. *Pitfalls*, besides the well-known side effects of frequently used drugs, include paediatric-specific concerns in terms of potential neurotoxicity in the developing brain and age-specific differences in drug distribution and metabolism. *Possibilities* to enhance our pharmacological understanding include the implementation of animal-based pharmacokinetic models that enable prediction of drug concentrations at target sites. This combined with multimodal neuromonitoring can link cerebral drug concentrations to drug effect and ultimately lead to more evidence-based analgosedation therapies in (paediatric) TBI.
